# Genetic Analysis of the ts-Lethal Mutant *Δpa0665*/*pTS-pa0665* Reveals Its Role in Cell Morphology and Oxidative Phosphorylation in *Pseudomonas aeruginosa*

**DOI:** 10.3390/genes15050590

**Published:** 2024-05-07

**Authors:** Jiayin Zhu, Hulin Zhao, Zhili Yang

**Affiliations:** Systems Biology, School for Marine Science and Technology, Zhejiang Ocean University, Zhoushan 316022, China; zhujiayin@zjou.edu.cn (J.Z.); hulin19982024@126.com (H.Z.)

**Keywords:** conditional allele, essential gene, Fe-S cluster, *Pseudomonas aeruginosa*

## Abstract

Pa0665 in *Pseudomonas aeruginosa* shares homologous sequences with that of the essential A-type iron–sulfur (Fe-S) cluster insertion protein ErpA in *Escherichia coli*. However, its essentiality in *P. aeruginosa* and its complementation with *E. coli erpA* has not been experimentally examined. To fulfill this task, we constructed plasmid-based *ts*-mutant *Δpa0665*/*pTS-pa0665* using a three-step protocol. The mutant displayed growth defects at 42 °C, which were complemented by expressing *ec.erpA*. Microscopic observations indicated a petite cell phenotype for *Δpa0665*/*pTS-pa0665* at 42 °C, correlated with the downregulation of the *oprG* gene. RNA sequencing revealed significant transcriptional changes in genes associated with the oxidative phosphorylation (OXPHOS) system, aligning with reduced ATP levels in *Δpa0665*/*pTS-pa0665* under 42 °C. Additionally, the *ts*-mutant showed heightened sensitivity to H_2_O_2_ at 42 °C. Overall, our study demonstrates the essential role of *pa0665* for OXPHOS function and is complemented by *ec.erpA*. We propose that the plasmid-based *ts*-allele is useful for genetic analysis of essential genes of interest in *P. aeruginosa*.

## 1. Introduction

*Pseudomonas aeruginosa*, a member of the *Pseudomonadaceae* family, is a Gram-negative, rod-shaped, motile bacterium that thrives in both aerobic and anaerobic conditions. It is widely found in nature, isolated from soil, plants, water, and animals, and is known for its versatility and opportunism [1]. The high mortality associated with *Pseudomonas* is mainly due to the emergence of drug-resistant strains, leading the WHO to prioritize it for research and new drug development [2].

Essential genes, which are crucial for microbial survival, are promising targets for drug development. Through the application of Tn-seq, Lee et al. identified 352 essential genes in *P. aeruginosa* [3], enriching the pool of potential targets for novel therapeutic interventions. Notably, 40 of these genes encode hypothetical proteins, whose functions and roles in cellular processes remain to be elucidated [3].

Hypothetical proteins, often identified across different lineages without functional validation, are a valuable resource for uncovering new biological insights and therapeutic targets [4,5]. The experimental analysis of these functionally unknown essential core genes will contribute to the understanding of new fundamental functions necessary for *Pseudomonas* growth [6]. This study is focused on *pa0665*, an essential gene coding for a hypothetical protein in *P. aeruginosa* PAO1. Comparative protein sequence analysis revealed that Pa0665 shares about 65.5% homologous sequences with ErpA, an A-type iron–sulfur (Fe-S) cluster insertion protein, deemed essential in *Escherichia coli* [7]; this is crucial for cellular respiration under aerobic conditions, as it contributes to the synthesis of isopentenyl diphosphate (IPP), a precursor for essential electron carriers like ubiquinone and menaquinone [8]. The absence of ErpA markedly diminishes complex I (NADH: ubiquinone oxidoreductase) content and NADH oxidase activity in the cytoplasmic membrane, significantly impacting the assembly of complex I [9], a testament to its essential role.

Despite the identified homology and the established essentiality of *erpA* in *E. coli* (*ec.erpA*), the functional importance of *pa0665* in *P. aeruginosa* and its potential complementation with *ec.erpA* has not been empirically verified. Addressing this gap, we constructed the plasmid-based temperature-sensitive (ts) mutant of *Δpa0665*/*pTS-pa0665* through our previously established three-step protocol [10,11]. We showed that *Δpa0665*/*pTS-pa0665* has growth defects at restrictive temperature (42 °C), reversible by expressing *ec.erpA*. Microscopic observations revealed a smaller cell phenotype for *Δpa0665*/*pTS-pa0665* at 42 °C, possibly linked to the downregulation of the *oprG* gene. RNA sequencing showed significant upregulation and downregulation of genes associated with cytochrome o ubiquinol oxidase and cytochrome c cbb3-type oxidase, indicating oxidative phosphorylation (OXPHOS) system disturbances in the *pa0665* ts-mutant, consistent with reduced ATP in *pa0665 ts*-mutant cells. Taken together, our results highlight the essential role of *pa0665* in the functioning of the OXPHOS system, which is complemented by *ec.erpA*. We propose that the plasmid-based *ts*-allele complementation presented here is a robust tool to unveil the molecular and biological function of unknown essential genes in *P. aeruginosa*.

## 2. Materials and Methods

### 2.1. DNA, Plasmids, and Bacterial Cultures

The oligonucleotides, plasmids, and bacterial strains used in this study are shown in Table 1. Strains were cultivated in LB (1 L: 10 g tryptone, 10 g NaCl, 5 g yeast extract, pH 7.0) liquid or solid medium supplemented with antibiotics (100 µg mL^−1^ ampicillin, 50 µg mL^−1^ gentamicin, and 100 µg mL^−1^ tetracycline) and chemicals (e.g., 10% sucrose or 0.2% arabinose) at 30 °C or 42 °C, as indicated.

### 2.2. Plasmid Construction

We used the same deletion plasmid and rescue plasmid (or ts plasmid) constructed in the previous study [10,11]. To construct the *pa0665* deletion and rescue plasmids, the deletion cassette and rescue cassette of *pa0665* were cloned into the deletion plasmid and rescue plasmid, respectively, using the ClonExpress II one-step cloning kit (Vazyme, Nanjing, China). Overexpression plasmids were constructed by cloning the araC-P_BAD_ promoter fragment and downstream gene fragment into the pBBR1MCS-5 plasmid [12] using the Vazyme cloning kit.

### 2.3. Plasmid-Based ts-Mutant Strain Construction

We used a three-step protocol that we developed previously [10,11] to construct the plasmid-based ts-lethal mutant strain *Δpa0665*/*pTS-pa0665*. Briefly, we first electroporated the deletion plasmid, which could not auto-replicate in *P. aeruginosa*, into the *P. aeruginosa* PAO1 strain, isolating integrants via a single crossover into the genome on a gentamicin-containing LB plate. Then, the rescue plasmid was transformed into the integrant on a tetracycline-containing plate. Subsequently, the counterselection of sacB generated the chromosomal *Δpa0665* allele by looping out the integrated plasmid on a sucrose-containing plate. The resulting strains were PCR validated for the chromosomal *Δpa0665* allele and assessed for ts-growth phenotype via spot-plating assay.

### 2.4. Spot-Plating Assay

The spot-plating assay [13] was employed to assess sensitivities to stress factors, including antibiotics, sucrose, hydrogen peroxide, and temperature. In brief, 10-fold serially diluted cultures were transferred onto LB plates supplemented with the relevant stress factors using a 48-pin replicator (V&P Scientific, Inc., San Diego, CA, USA) and incubated at 30 °C or 42 °C as required.

### 2.5. Fluorescence Microscopic Analysis

Cell morphology was investigated under the Olympus BX53 microscope (Olympus, Tokyo, Japan) using the phase contrast configuration. Nile red fluorescent dye was used to visualize the cytoplasmic membrane.

### 2.6. Fluorescence Activated Cell Sorting (FACS) Analysis

Cells were fixed with 70% ethanol for 30 min and washed with PBS 3 times. We utilized the green fluorescent dye PicoGreen (Solarbio, Beijing, China) to distinguish bacterial cells from other small particulate impurities in the liquid. Fixed cells were resuspended in PBS to a final concentration of OD_600_ = 0.6, and the green dye, PicoGreen, was added. The PicoGreen concentrated solution provided by the supplier was diluted 1:200 in dimethyl sulfoxide (DMSO) and added to cells at a ratio of 5 μL of diluted dye to 100 μL of cells. The cells were stained for 30 min at room temperature and diluted with 1 mL of PBS containing a 1:1000 dilution of PicoGreen. Stained cells were subjected to FACS analysis using Becton Dickinson FACS Calibur (BD Biosciences, San Diego, CA, USA) with a 488 nm laser. Data were processed with CellQuest software (version 5.1; BD Biosciences). 

### 2.7. ATP Content Measurement

Cells were centrifuged at 10,000× *g* for 5 min at 4 °C. The pellets were treated with a lysis buffer from an ATP detection kit (Beyotime, Haimen, China) for 1 min at room temperature and then were centrifuged at 10,000× *g* for 5 min. The supernatant was transferred to a new 1.5 mL tube for an ATP test with the ATP detection kit purchased from Beyotime (China). The relative ATP content was determined using the formula: relative ATP content = ATP value/protein value. Protein concentration in the sample was quantified using a Bradford 1× Dye Reagent (Bio-Rad, Hercules, CA, USA), measured at a wavelength of 595 nm.

### 2.8. RNA Extraction and RNA-Seq Analysis

Total RNA was extracted in triplicate from four cell samples (wt and ts-mutant strains, incubated for 6 h at 30 °C and 42 °C after the second subculture) using a TaKaRa Bio Inc. RNA extraction kit and quality-checked with an Agilent 2100 Bioanalyzer. Afterward, samples underwent DNase I treatment (TaKaRa) and rRNA removal with an Epicentre Biotechnologies Ribo-Zero magnetic kit. The resulting rRNA-depleted RNA underwent RNA-seq on an Illumina HiSeq 2500 at the Shanghai Human Genome Centre (Shanghai, China) using paired-end (PE150) sequencing. For library construction, 100 ng of RNA was used with the NEB Next Ultra Directional RNA library prep kit. Data analysis was carried out on the RaNA-Seq [14] cloud platform, with raw data cleaned using Fastp software (version 0.2) [15] and mapped to the reference genome (Pseudomonas Genome Database version 22.1; www.pseudomonas.com accessed on 15 March 2024) using salmon [16]. Gene expression was normalized to TPM (transcripts per kilobase million), with differentially expressed genes (DEGs) identified using DEseq2 [17], based on a >4-fold change and *p* value < 0.01.

### 2.9. Statistics

Data are presented as mean ± standard error. The statistical significance of differences was assessed using an unpaired, two-tailed Student’s *t*-test. A *p*-value < 0.05 was deemed statistically significant.

### 2.10. Data Availability

The RNA-seq raw data sets were submitted to NCBI with the accession numbers PRJNA1085980 for wt and ts mutant at 30 °C and 42 °C.

## 3. Results

### 3.1. pa0665 Gene Is Essential for Growth on LB-Agar Plate

**Figure 1:**
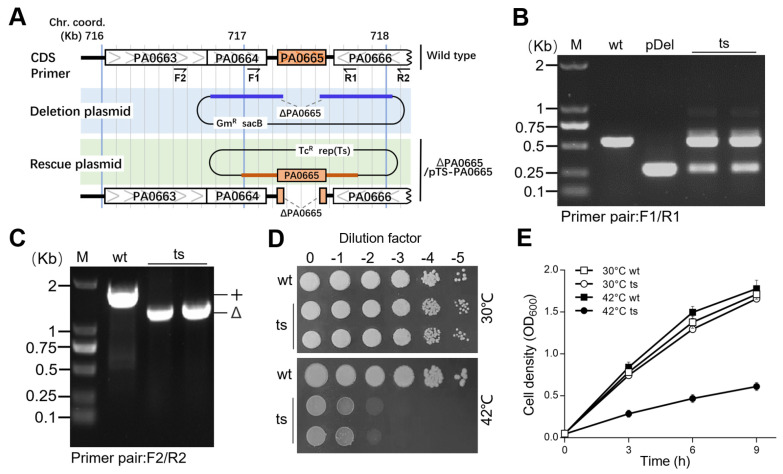
*Δpa0665*/*pTS-pa0665* exhibits growth defect on LB at the restrictive temperature. (**A**) The *pa0665* deletion allele cassette (blue line) in the deletion plasmid and complementary sequences (brown line) in the rescue plasmid are shown in a physical map. *Δpa0665*/*pTS-pa0665* (ts) carries a deletion allele, *Δpa0665*, on the chromosome, along with a complementary copy of *pa0665* controlled by a native promoter on a temperature-sensitive plasmid. (**B**,**C**) PCR assays for *pa0665* alleles use F1-R1 and F2-R2 primer pairs located inside and outside the *pa0665* complementary sequences on the rescue cassette, revealing the chromosomal deletion allele in *Δpa0665*/*pTS-pa0665* isolates. (**D**) Spot-plating assay displays *Δpa0665*/*pTS-pa0665* growth, where 10-fold serial dilutions of wild-type and mutant cells were spotted on an LB plate and incubated overnight at 30 °C and 42 °C. (**E**) Growth curves of the second subcultures are shown, with time (h) on the *x*-axis and cell density (OD_600_) on the *y*-axis.

The construction of bacterial strains lacking essential genes typically led to the emergence of suppressors [18]. To circumvent this issue, we removed the gene *pa0665* from the chromosome while keeping a complimentary copy in a temperature-sensitive (ts) suicide plasmid. Using a detailed three-step process [10,11], we successfully engineered a chromosomal deletion of *pa0665* (*Δpa0665*), safeguarded by an identical, plasmid-based *pa0665* gene under the control of its native promoter in plasmid pTS*−pa0665* (Figure 1A). The *Δpa0665*/*pTS-pa0665* ts-mutant strain was validated using PCR with primers F1/R1 and F2/R2 (Figure 1A), where F1/R1 sites are present, and F2/R2 sites are absent in the complementary copy of the rescue plasmid pTS-*pa0665* (Figure 1B,C). Spot-plating assays showed that *Δpa0665*/*pTS-pa0665* experienced reduced growth at 42 °C compared to the wild type, which displayed similar growth patterns at 30 °C (Figure 1D). These findings affirm *pa0665*’s essential role in growth in LB medium. Removing the high-copy ts-plasmid from the mutant’s cells would take several generations at higher temperatures; hence, we developed a sequential subculturing approach, reducing plasmid numbers in the first stage and allowing phenotype expression in the second subculture as described in our previous work [11]. The second subculture showed the growth defect of *Δpa0665*/*pTS-pa0665* at 42 °C (Figure 1E).

### 3.2. Putative Ortholog erpA in E. coli Functionally Complements the Defect of pa0665 in P. aeruginosa

**Figure 2:**
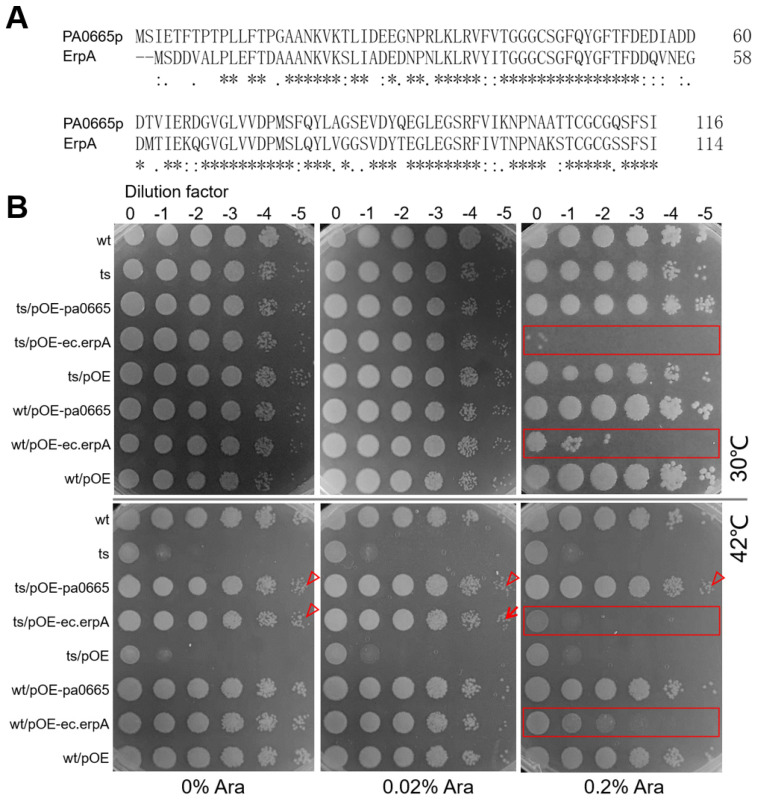
Putative ortholog *erpA* from *E. coli* rescues the growth defect of *Δpa0665*/*pTS-pa0665* at 42 °C. (**A**) Protein sequence alignment between Pa0665 and ErpA. Asterisk (*) indicates positions where residues are identical across all sequences; Colon (:) indicates conservation between groups of strongly similar properties; Dot (.) indicates conservation between groups of weakly similar properties. (**B**) Spot-plating assay. No induction (0 arabinose) or mild induction (0.02% arabinose) of *erpA-OE* rescues the growth defect of *Δpa0665*/*pTS-pa0665* at 42 °C (see down row arrowhead and arrow). No mild and strong induction (0.2% arabinose) of *pa0665*-OE rescues the growth defect of *Δpa0665*/*pTS-pa0665* at 42 °C (see up row arrowheads). Strong induction of *erpA*-OE hampers the growth of *Δpa0665*/*pTS-pa0665* and wild-type cells (see rectangles); wt/pOE and ts/pOE served as the plasmid control.

Using BLASTp alignment (www.ncbi.nlm.nih.gov/blast accessed on 15 March 2024), we discovered that the protein sequences of Pa0665 from *P. aeruginosa* and ErpA from *E. coli* share 65.5% identity across 116 overlapping residues (Figure 2A). This level of identity, exceeding 40%, is sufficient to suggest a structural similarity between the two proteins [19]. To determine whether *ec.erpA* is a true ortholog of *pa0665* in *P. aeruginosa*, a complementation experiment was performed. We engineered overexpression constructs for *pa0665*-OE and *ec.erpA*-OE, with transcription regulated by the arabinose-inducible P_BAD_ promoter [20] in the multi-host pBBR1MCS-5 plasmid [12], resulting in pOE-*pa0665* and pOE-*ec.erpA* constructs. Spot-plating assay indicated that under no inducer arabinose, multi-host plasmid pOE-*ec.erpA* at leakage expression level was sufficient to rescue the growth defect of *Δpa0665*/*pTS-pa0665* at 42 °C, similar to the positive control plasmid pOE-pa0665 (Figure 2B, see arrowheads). Mild induction of *ec.erpA* with 0.02% arabinose rescued the growth defect of the *Δpa0665*/*pTS-pa0665 pOE-ec.erpA* strain at 42 °C (Figure 2B, see arrow), while strong induction of *ec.erpA* with 0.2% arabinose impeded the growth of *Δpa0665*/*pTS-pa0665 pOE-erpA* strain and the wild type (Figure 2B, see rectangles). These findings confirm that leaky expression and mild induction of *ec.erpA* can functionally compensate for the growth defect caused by *pa0665* deficiency in *P. aeruginosa*. 

### 3.3. The Δpa0665/pTS-pa0665 Mutant Exhibits Petite Cell Morphology under Restrictive Temperature

**Figure 3:**
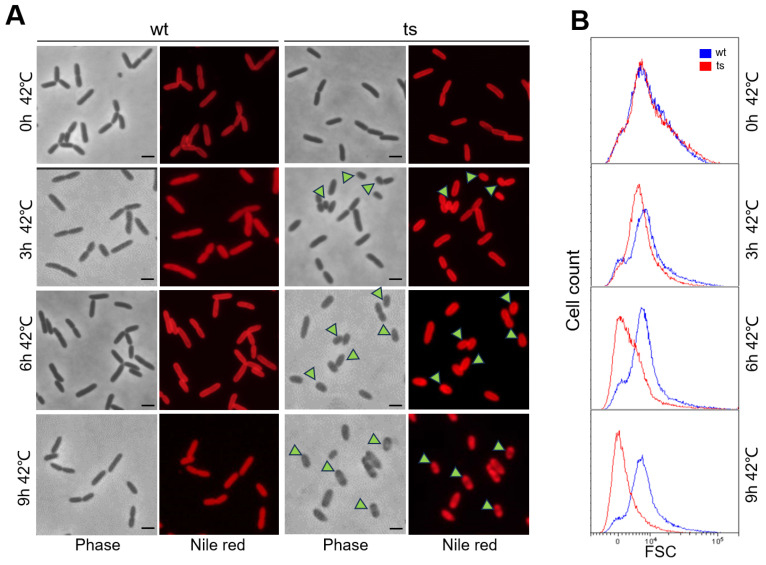
*Δpa0665*/*pTS-pa0665* exhibits petite cell morphology under restrictive temperature. (**A**) The cells were examined prior to the temperature shift from 30 °C to 42 °C during the first subculture or at 0 h, 3 h, 6 h, and 9 h after the second subculture began at 42 °C. Petite cell morphology is indicated by arrowheads, with a 1 μm scale bar shown. (**B**) FACS analysis of cell size, with the *x*-axis representing cell size and the *y*-axis indicating cell count.

To investigate the impact of *pa0665* depletion on cell morphology, we examined the terminal phenotype of *Δpa0665*/*pTS-pa0665* at 42 °C. Both the mutant and wild-type strains underwent a temperature shift from 30 °C to 42 °C. Samples from the second subculture at 0 h, 3 h, 6 h, and 9 h at 42 °C were fixed and stained with Nile red for fluorescence microscopy. At 0 h, *Δpa0665*/*pTS-pa0665* displayed a wild-type-like rod-shaped morphology (Figure 3A, top row). At 3 h and 6 h, the mutant cells exhibited a petite phenotype (Figure 3A, middle two rows, see arrowheads). By 9 h, ghost cells or lysed cells began to appear alongside petite cells (Figure 3A, bottom row, see arrowheads). After growth for 9 h in the second subculture, in addition to the petite cells, ghost cells or lysed cells started to appear (Figure 3A, bottom row, see arrowheads). Fluorescence-activated cell sorting (FACS) analysis corroborated the presence of the petite phenotype in *Δpa0665*/*pTS-pa0665* cells under 42 °C (Figure 3B). These findings suggest that the depletion of *pa0665* in *P. aeruginosa* leads to a significant reduction in cell size.

### 3.4. ATP Content Was Decreased in Δpa0665/pTS-pa0665 Mutant under Restrictive Temperature

**Figure 4:**
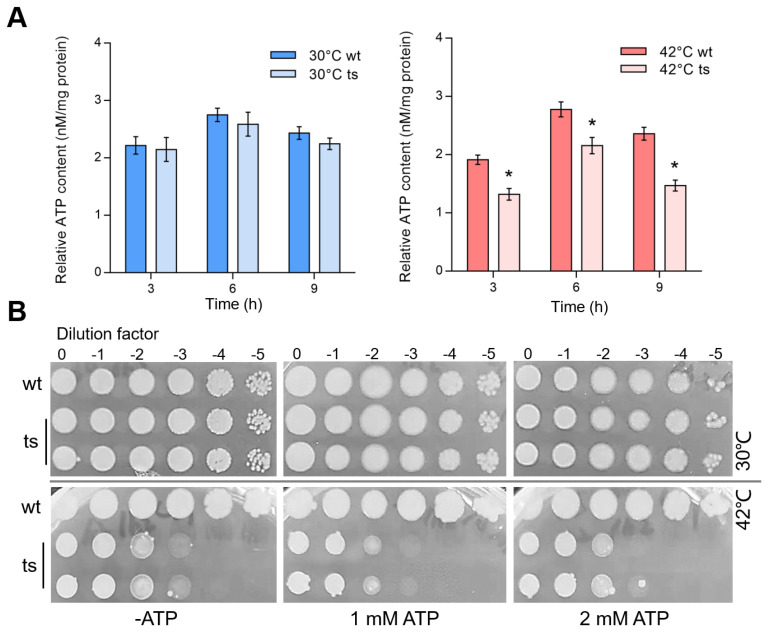
Reduced ATP levels in *Δpa0665*/*pTS-pa0665* mutant at 42°C and exogenous ATP fails to rescue lethal phenotype. (**A**) Intracellular ATP Level after the temperature shift to 42 °C compared to that at 30 °C in *Δpa0665*/*pTS-pa0665* (ts) and wild-type (wt) cells. Cells for ATP assays were collected at 3 h, 6 h, and 9 h after the second subculture was started at 42 °C. *, *p* < 0.05; n = 3. (**B**) Spot-plating assay shows that supplementing with 1 mM ATP or 2 mM ATP concentrations does not compensate *Δpa0665*/*pTS-pa0665* growth defect at 42 °C.

Fe-S cluster insertion proteins were required for an oxidative phosphorylation (OXPHOS) system that generates energy molecule ATP for cell growth. To evaluate whether the *pa0665* deficiency impacts cellular ATP levels, we measured the ATP content in the *Δpa0665*/*pTS-pa0665* mutant at 42 °C. The results showed that at 3 h, 6 h, and 9 h of incubation at 42 °C after a second subculture, the *Δpa0665*/*pTS-pa0665* mutant exhibited a significant reduction in ATP content, which was not observed at 30 °C, indicating a temperature-sensitive phenotype (Figure 4A). In addition, we evaluated whether supplementing ATP could rescue the growth defect of *Δpa0665*/*pTS-pa0665* at 42 °C. Serial dilutions of cultures were spot-plated on LB-agar supplemented with 0, 1 mM, or 2 mM ATP. Contrary to our expectations, exogenous ATP with 1 mM or 2 mM did not restore the growth of the *Δpa0665*/*pTS-pa0665* mutant at 42 °C (Figure 4B), suggesting that the growth defect at the restrictive temperature is not simply due to ATP depletion but likely involves more complex metabolic disturbances. 

### 3.5. The Δpa0665/pTS-pa0665 Mutant Is Hypersensitive to Oxidative Stress Mediated by H_2_O_2_

**Figure 5:**
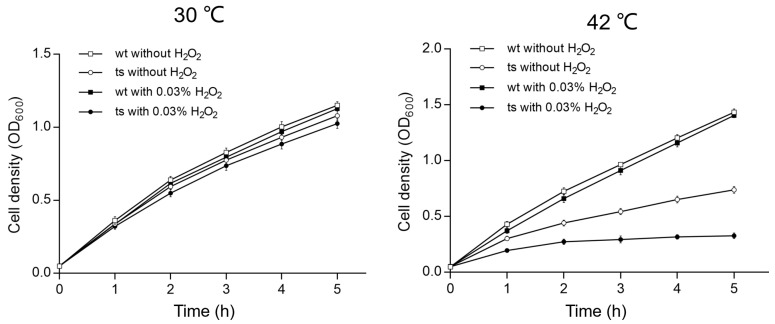
Sensitivity of the *Δpa0665*/*pTS-pa0665* mutant to H_2_O_2_ compared to wild type. Growth curves of *Δpa0665*/*pTS-pa0665* (ts) and wild-type (wt) at 30 °C and 42 °C after second subcultures. The x and y axes show the time (h) treated with or without H_2_O_2_ and cell density (OD_600_), respectively.

Iron–sulfur (Fe-S) cluster proteins are critical for a variety of cellular processes, including electron transfer, enzyme activation, and the regulation of gene expression [21,22]. These proteins play a significant role in antioxidant activities by facilitating redox reactions and protecting cells from oxidative damage [23]. To evaluate the oxidative stress tolerance of the *Δpa0665*/*pTS-pa0665* mutant at 42 °C, we conducted a growth curve experiment over a period of 5 h to assess bacterial sensitivity to hydrogen peroxide (H_2_O_2_). This duration was strategically chosen considering the known degradation of H_2_O_2_ over time. Since H_2_O_2_’s most pronounced effects occur shortly after its application, we standardized the initial OD_600_ of all strain samples, ensuring an equal oxidative challenge during H_2_O_2_’s effective period. We exposed both the wild-type and *Δpa0665*/*pTS-pa0665* strains to 0.03% H_2_O_2_ in LB medium for 5 h at 30 °C and 42 °C following a second subculture. Upon exposure to 0.03% H_2_O_2_, both strains demonstrated a minor growth decline (Figure 5). Notably, at 42 °C, the *Δpa0665*/*pTS-pa0665* mutant displayed a pronounced vulnerability to H_2_O_2_, indicating a higher sensitivity at 42 °C in comparison to 30 °C (Figure 5). These findings suggest that the presence of *pa0665* is critical for protection against oxidative stress caused by H_2_O_2_.

### 3.6. Transcriptomic Analysis Reveals Impaired Oxidative Phosphorylation in pa0665-Deficient P. aeruginosa 

**Figure 6:**
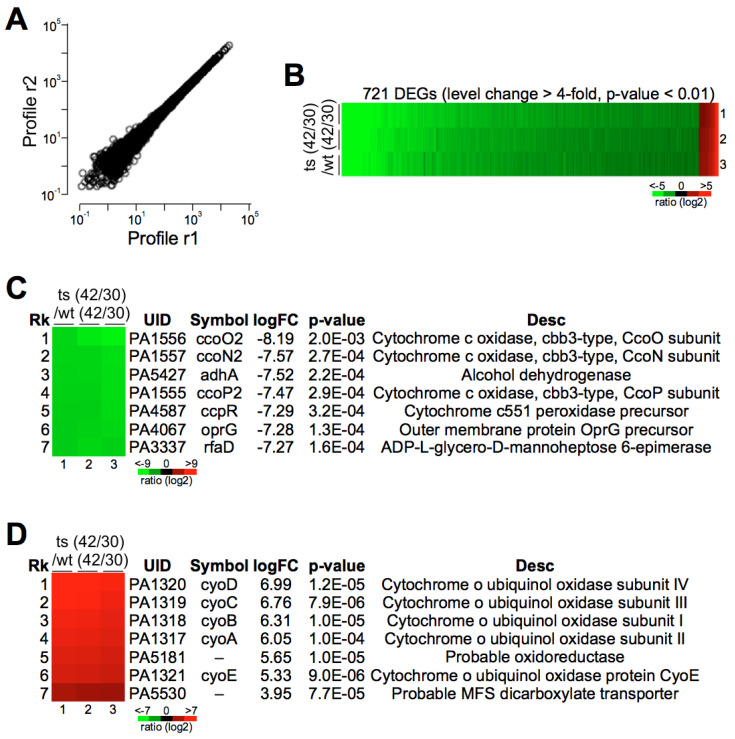
Transcriptional changes in *Δpa0665*/*pTS−pa0665* mutant compared to wild-type at 42°C and 30 °C. (**A**) Reproducibility of samples. The scatter plot represents the correlation of gene expression data. The x and y axes, respectively, represent the gene expression levels that were randomly taken from two technical replicates, illustrating a high degree of linear correlation indicative of good reproducibility. (**B**) Heatmap of differentially expressed genes (DEGs). Displaying 721 genes identified with differential expression (fold change > 4, *p*-value < 0.01) between the *Δpa0665*/*pTS-pa0665* and wild-type strains. The color gradient in the heatmap corresponds to the log_2_ fold change in expression levels, ranging from −5 (red) to +5 (green). (**C**,**D**) Top 7 most upregulated or downregulated genes in the *Δpa0665*/*pTS-pa0665* compared to the wild-type.

To elucidate the impact of *pa0665* deletion on the gene expression profile of *P. aeruginosa* and identify genes potentially associated with oxidative phosphorylation, we performed transcriptome sequencing (RNA-seq) analysis under both 30 °C and 42 °C for the wild type and the *Δpa0665*/*pTS-pa0665* mutant in triplicate (Figure 6A). This comprehensive analysis revealed 721 genes with significant transcriptional changes between the two temperatures in the *Δpa0665*/*pTS-pa0665* (ts) mutant, diverging markedly from the wild type (Rts/Rwt change > 4-fold, FDR-adjusted *p*-value < 0.01, n = 3) (Figure 6B). Notably, the top differentially expressed genes include those encoding components of the cytochrome o ubiquinol oxidase and cytochrome c cbb3-type oxidase (Figure 6C,D), underscoring a substantial disturbance in the oxidative phosphorylation (OXPHOS) system of the mutant. These findings suggest that *pa0665* plays a critical role in maintaining OXPHOS functionality.

### 3.7. Impairment of pa4067/oprG Possibly Linked to the Altered Morphology of Δpa0665/pTS-pa0665 Mutant at 42 °C

**Figure 7:**
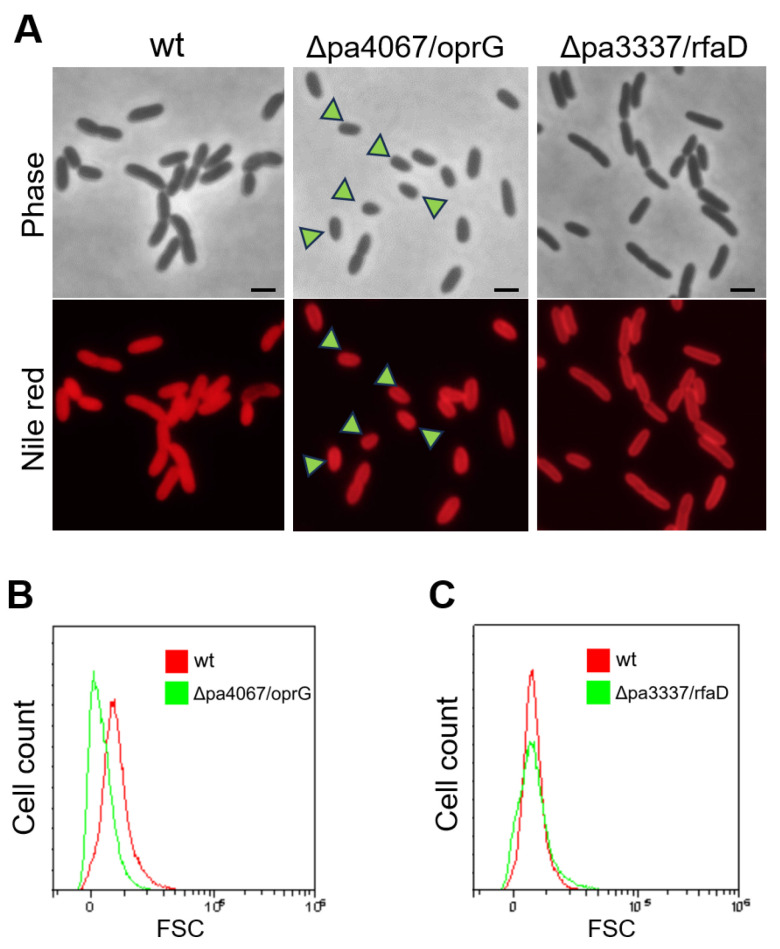
Cellular morphology analysis of *pa4067*/*oprG* and *pa3337*/*rfaD* mutants. (**A**) Fluorescence microscopy analysis of *Δpa4067*/*oprG* and *Δpa3337*/*rfaD* mutant strains. Arrowheads indicate the petite cell morphology. A scale bar of 1 μm is shown. (**B**,**C**) FACS analysis of cell size. X and Y axes indicate cell size and cell count, respectively.

Transcriptomic analyses revealed that *pa3337*/*rfaD* and *pa4067*/*oprG* were among the top seven significantly downregulated genes (Figure 6C), both critical for the structural integrity of the bacterial outer membrane. *oprG* plays a key role in forming outer membrane channels in Gram-negative bacteria, crucial for molecular transport and affecting bacterial virility and antibiotic resistance [24], while *rfaD* is vital for lipopolysaccharide (LPS) biosynthesis, a key outer membrane component in Gram-negative bacteria [25,26]. To investigate the impact of disrupting *pa3337*/*rfaD* and *pa4067*/*oprG* on cellular morphology, we constructed knockout plasmids containing sequences approximately 500 bp identical to the N-terminal coding sequences of *pa3337*/*rfaD* and *pa4067*/*oprG*. These plasmids were then inserted into the corresponding genes of the wild-type *P. aeruginosa* strain through homologous recombination, creating knockout mutants for *pa3337*/*rfaD* and *pa4067*/*oprG*. Fluorescence microscopy revealed a decrease in cell size for the *pa4067*/*oprG* mutants compared to wild-type cells (Figure 7A). In contrast, cell size in *pa3337*/*rfaD* mutants did not differ significantly from that of wild-type cells, a conclusion supported by flow cytometry analysis (Figure 7B,C). These findings suggest that mutations in *pa4067*/*oprG* lead to decreased cell size, indicating a potential connection between diminished *oprG* expression and the smaller cell size observed in *Δpa0665*/*pTS-pa0665* mutants at 42 °C.

## 4. Discussion

This study contributed to the understanding of *pa0665*, a hypothetical protein in *P. aeruginosa*, by building on foundational work that identified essential genes in the PAO1 strain of *P. aeruginosa*, including *pa0665* [3]. Our investigation provides insights into the functional significance of this gene, which was predicted to be essential but had not been experimentally validated. We constructed the plasmid-based temperature-sensitive (ts) lethal mutant strain, *Δpa0665*/*pTS-pa0665*, using a three-step protocol that we previously developed [10,11]. This approach enabled us to suggest the importance of *pa0665* for the growth and survival of *P. aeruginosa* (Figure 1) and to conduct a functional complementation analysis with *ec.erpA*, a key A-type iron–sulfur (Fe-S) cluster insertion protein [8], and to validate their orthologship (Figure 2). The functional complementation by *ec.erpA* indicates a potential evolutionary conservation between these proteins and hints at common vulnerabilities in bacteria that might be of interest for further research in antimicrobial strategies.

Iron–sulfur (Fe–S) proteins are crucial for prokaryotic and eukaryotic cell metabolism [21,27,28]. In *E. coli*, *erpA* is involved in various metabolic pathways, including respiratory metabolism [8,9,29]. Our transcriptomic analysis of the *pa0665* temperature-sensitive mutant revealed changes in gene expression related to the oxidative phosphorylation system, including alterations in the expression of genes involved in the electron transport chain (Figure 6), mainly cytochrome o ubiquinol oxidase and cytochrome c cbb3-type oxidase, that participate in the electron transport chain’s final steps [30,31]. These alterations align with the observed growth defects and reduced ATP levels (Figure 4) of *Δpa0665*/*pTS-pa0665* under 42 °C, suggesting a possible role of *pa0665* in oxidative phosphorylation functionality and cellular energy metabolism.

In response to oxidative stress, like exposure to hydrogen peroxide, *E. coli* activates defense mechanisms, including the expression of antioxidant enzymes. For example, the *isc* operon, which specifies Fe-S cluster formation and repair activities, is known to be induced by hydrogen peroxide, independent of the common oxidative stress regulators OxyR and SoxRS [32]. This suggests a direct link between Fe-S cluster biogenesis and the cellular response to oxidative stress. In this work, we found that the *Δpa0665*/*pTS-pa0665* mutant displayed heightened sensitivity to oxidative stress under restrictive temperature, indicating a potential role for *pa0665* in protecting against oxidative damage. It seems that disruption of *pa0665* might impact Fe-S cluster assembly or repair, affecting enzymes involved in detoxifying reactive oxygen species. 

Additionally, our results indicated a reduction in cell size associated with *pa0665* depletion (Figure 3), correlated with the downregulation of *oprG* in our transcriptomic analysis (Figure 6), a gene encoding a major outer membrane protein [24,33]. This observation suggests a role for *pa0665* in maintaining not only metabolic processes but also cellular structure and integrity.

In summary, while our study contributed to the understanding of the role of *pa0665* in *P. aeruginosa*, it also highlighted areas for future investigation. These included its function in energy metabolism, oxidative stress response, and cellular integrity. The insights provided could contribute to the broader field of research on hypothetical proteins and essential genes and may inform future developments in antimicrobial therapies. The methodology used in this work, particularly the plasmid-based *ts*-allele approach, might offer a useful framework for genetic analysis in understanding the roles of essential genes in bacterial biology.

## 5. Conclusions

This study provided a foundational understanding of the *pa0665* gene in *P. aeruginosa*, elucidating its significant role in cell morphology and oxidative phosphorylation. Our findings revealed that the *Δpa0665*/*pTS-pa0665* mutant exhibits a distinct petite cell phenotype and altered ATP production under restrictive temperatures (42 °C). The lethal phenotype caused by the deletion of *pa0665* can be reversed through the expression of the homologous *ec.erpA* gene, which underscores the potential functional similarities between these organisms. However, the specific mechanisms by which *pa0665* influences cellular processes in *P. aeruginosa* require further investigation. This study contributed to the broader understanding of essential genes in bacteria and offers a basis for future research into the complex biology of *P. aeruginosa*, with potential implications for targeted antibiotic development.

## Figures and Tables

**Table 1:** Oligonucleotides, plasmids, and strains used in this study.

Oligonucleotides		
Name	Sequence (5′-3′)	Usage
F1	CTGGAACTGCCTGCCAGCGT	Assay pa0665 alleles in chr and TS plasmid
R1	CGGCAACTGCCCTGATGTGA	Ditto
F2	CTCCGGCATTTCCAGTCGAT	Assay pa0665 alleles in chr but not TS plasmid
R2	AGGTGAACCACGCACTGCTG	Ditto
Plasmids	Relevant genotype	Reference
pDEL	pUC-Gm^r^-sacB	[10,11]
pRES or pTS	pUC-Tc^r^-ori^ts^	[10,11]
pOE	pBBRMCS-5-araC-P_BAD_-Gm^r^	[10,11]
pDEL-pa0665	pa0665 deletion cassette in pDEL	This study
pRES-pa0665	pa0665 rescue cassette in pTS	This study
pOE-pa0665	araC-P_BAD_- pa0665 in pOE	This study
pOE-ec.erpA	araC-P_BAD_-ec.erpA in pOE	This study
Strains	Rel genotype/Usage	Reference
PAO1	Wild type	[10,11]
Δpa0665/pTS-pa0665	pa0665 ts-allele	This study
Δpa0665/pTS-pa0665/pOE-pa0665	pa0665-OE in ts	This study
Δpa0665/pTS-pa0665/pOE-ec.erpA	ec.erpA-OE in ts	This study
Δpa0665/pTS-pa0665/pOE	pOE in ts	This study
Δpa4067/oprG	pa4067-deletion	This study
Δpa3337/rfaD	pa3337-deletion	This study
wt/pOE-pa0665	pa0665-OE in wt	This study
wt/pOE-ec.erpA	ec.erpA-OE in wt	This study
wt/pOE	pOE in wt	This study

## Data Availability

The original contributions presented in the study are included in the article, further inquiries can be directed to the corresponding author.
